# TREM2^+^ Macrophages Promote Immune Evasion in Endometriosis Through BAG3‐Associated Regulation of CD8^+^ T Cell Autophagy

**DOI:** 10.1002/advs.78385

**Published:** 2026-11-12

**Authors:** Ming Yuan, Xue Jiao, Le Xu, Qianhui Ren, Shuang Wang, Zhaoyang Zhong, Na Li, Qiuju Li, Xiaoli Meng, Guoyun Wang

**Affiliations:** ^1^ Department of Obstetrics and Gynecology Shandong Provincial Hospital Affiliated to Shandong First Medical University Jinan Shandong China; ^2^ Department of Obstetrics and Gynecology Shandong Provincial Hospital Affiliated to Shandong University Jinan Shandong China; ^3^ Shandong Key Laboratory of Reproductive Research and Birth Defect Prevention Shandong First Medical University Jinan Shandong Province China; ^4^ Shandong Provincial Health Commission Key Laboratory of Prevention and Treatment for Major Gynecological Diseases Shandong Provincial Hospital Affiliated to Shandong First Medical University Jinan Shandong Province China; ^5^ JiNan Key Laboratory of Diagnosis and Treatment of Major Gynecological Disease Shandong Provincial Hospital Affiliated to Shandong First Medical University Jinan Shandong Province China; ^6^ Gynecology Laboratory Shandong Provincial Hospital Affiliated to Shandong First Medical University Jinan Shandong Province China; ^7^ Gynecology Laboratory Medical Science and Technology Innovation Center Shandong First Medical University & Shandong Academy of Medical Sciences Jinan Shandong Province China; ^8^ Lc‐Bio Technologies (Hangzhou) Co., Ltd Hangzhou Zhejiang China

**Keywords:** BAG3, endometriosis, immune dysfunction, macrophage, single‐cell RNA sequencing, TREM2

## Abstract

Macrophages are the most abundant immune cells in endometriotic lesions, but the roles of specific macrophage subsets in local immune dysfunction remain poorly understood. Here, combining single‐cell RNA sequencing of 11 clinical samples with TREM2 knockout mouse models, co‐culture experiments, and pharmacological intervention, we identify TREM2^+^ macrophages as an immunoregulatory population enriched in endometriosis. CD8^+^ T and natural killer (NK) cells in endometriotic lesions exhibited exhaustion‐associated features and reduced cytotoxicity. Cell–cell communication analysis and functional experiments revealed that TREM2^+^ macrophages regulated CD8^+^ T cell activity through SPP1‐CD44‐associated communication and BAG3‐associated autophagy regulation. In a mouse model of endometriosis, TREM2 deficiency enhanced CD8^+^ T and NK cell cytotoxicity and reduced ectopic lesion growth. Pharmacological disruption of the BAG3–HSP70 interaction with YM‐1 impaired CD8^+^ T cell function and promoted lesion growth, and BAG3 silencing reduced autophagy‐related markers and effector function in CD8^+^ T cells, supporting the involvement of BAG3‐associated regulation in TREM2^+^ macrophage‐mediated immune dysfunction. Our data identify TREM2^+^ macrophages as important regulators of immune dysregulation in endometriosis and highlight TREM2 as a potential target for future non‐hormonal therapeutic strategies.

## Graphical abstract

TREM2^+^ macrophages are enriched in endometriosis and suppress CD8^+^ T cell function through SPP1–CD44 communication and BAG3‐associated autophagy regulation. Genetic TREM2 deficiency restores cytotoxic lymphocyte activity, whereas antibody‐mediated TREM2 blockade reduces ectopic lesion growth, highlighting TREM2 as a potential non‐hormonal therapeutic target.

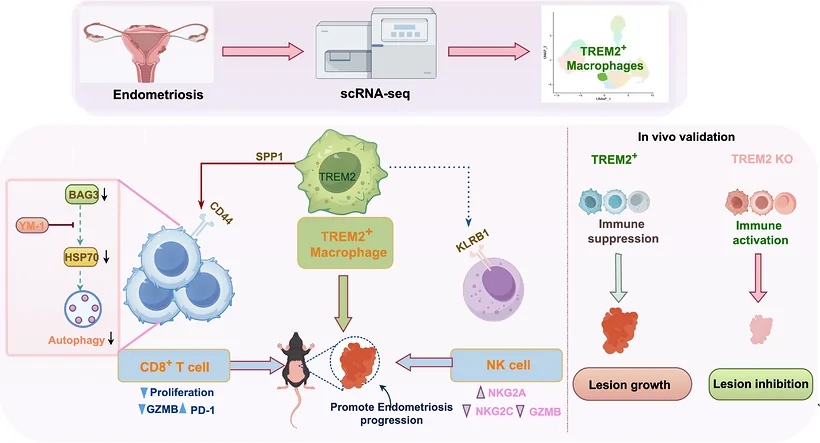

## 1 Introduction

Endometriosis is an estrogen‐dependent inflammatory disorder characterized by the growth of endometrium‐like tissue outside the uterus, leading to chronic pelvic pain and infertility [1]. It affects approximately 200 million women of reproductive age globally, and is present in roughly 50% of women with chronic pelvic pain and 30–50% of those with infertility [2]. Endometriosis has been associated with a 4.2‐fold higher risk of ovarian cancer [3]. The pathogenesis of endometriosis remains incompletely understood, although accumulating evidence suggests that the immune system plays a central role [4]. Despite the clinical benefits of hormonal therapies, recurrence and incomplete responses remain major challenges, highlighting the importance of identifying non‐hormonal therapeutic targets.

Macrophages, which originate from embryonic progenitors and bone marrow‐derived monocytes, are essential for tissue homeostasis and host defense [5]. Macrophages display considerable functional plasticity, as their phenotypes and effector functions are shaped by signals from the local microenvironment, which can accelerate or inhibit disease progression [6]. In addition, macrophage subsets can suppress lymphocyte responses through various mechanisms [7]. In endometriotic lesions, macrophages are among the most abundant immune cells and have been shown to promote lesion growth, vascularization, and innervation [8]. Yet the specific macrophage subsets that drive immune evasion in endometriosis have not been defined.

CD8^+^ T cells and natural killer (NK) cells are important cytotoxic lymphocyte populations in the endometriotic microenvironment, and functional alterations with impaired cytotoxicity have been reported in endometriosis [9]. Single‐cell transcriptomic studies have revealed enhanced crosstalk between macrophages and lymphocytes in endometriosis, which may contribute to local immunosuppression [10]. These observations suggest that specific macrophage subsets actively drive CD8^+^ T and NK cell dysfunction, although the mechanisms involved are not clear.

Among macrophage subsets, those expressing triggering receptor expressed on myeloid cells 2 (TREM2) have attracted attention for their roles in diverse diseases, including neurodegeneration and multiple malignancies such as glioblastoma and renal cell carcinoma [11, 12, 13, 14]. The function of TREM2, however, appears to be context‐dependent. In glioblastoma, TREM2 expression is negatively associated with immunosuppressive myeloid signatures and T cell exhaustion [15], whereas in hepatocellular carcinoma, TREM2 exerts protective effects through pleiotropic mechanisms [16]. More generally, TREM2 upregulation in tumor‐associated macrophages promotes disease progression through metabolic reprogramming and immunosuppression [17, 18]. Although TREM2^+^ macrophages have been detected in endometriotic tissue [19], their function in the local immune microenvironment has not been characterized.

Here, we performed integrated single‐cell RNA sequencing (scRNA‐seq) of endometriotic lesions and peritoneal fluid (PF) samples from patients with endometriosis, as well as simple cysts and peritoneal fluid samples from patients without endometriosis. We aimed to characterize endometriosis‐associated macrophage heterogeneity, focusing on the TREM2^+^ subset and its role in local immunosuppression.

## 2 Results

### 2.1 Single‐Cell Transcriptomic Profiling of the Cellular Landscape in Endometriosis

To characterize the cellular composition of endometriosis, we performed scRNA‐seq on the 10x Genomics platform to analyze 11 samples from eight individuals, including four PF and three endometriotic lesions from five patients with endometriosis, as well as two simple cysts and two PF samples from patients without endometriosis (Figure 1A and Table S1). After removing ambient RNA contamination and quality filtering, we included 83,572 cells for downstream analysis. Following data integration, we applied uniform manifold approximation and projection (UMAP) for dimensionality reduction. Unsupervised clustering revealed 16 clusters representing the major cell populations within the endometriotic microenvironment (Figures 1B and S1A), including macrophages (CD14^+^ and CD163^+^), dendritic cells (DC‐CD1C: CD1C^+^; DC‐CLEC9A: CLEC9A^+^), mast cells (KIT^+^), T cells (CD3D^+^), NK cells (NKG7^+^), B cells (CD79A^+^), plasma cells (IGHG1^+^), endothelial cells (VWF^+^), pericytes (MYH11^+^), fibroblasts (DCN^+^), and neutrophils (FCGR3B^+^), each defined by canonical markers (Figures 1D and S1B) [20, 21]. All cell types were detected across patients and sample types, although their proportions differed. Tissue samples were enriched for fibroblasts, whereas macrophages and T cells predominated in PF (Figures 1C,E and S1C). Flow cytometric analysis of ectopic tissues (*n* = 25) and PF (*n* = 18) from patients with endometriosis, as well as cyst walls (*n* = 14) and PF (*n* = 10) from control subjects, validated the scRNA‐seq findings (Figures 1F and S1D). Functional enrichment analysis showed that immune cell subtypes had different pathway profiles (Figure S1E).

**FIGURE 1:**
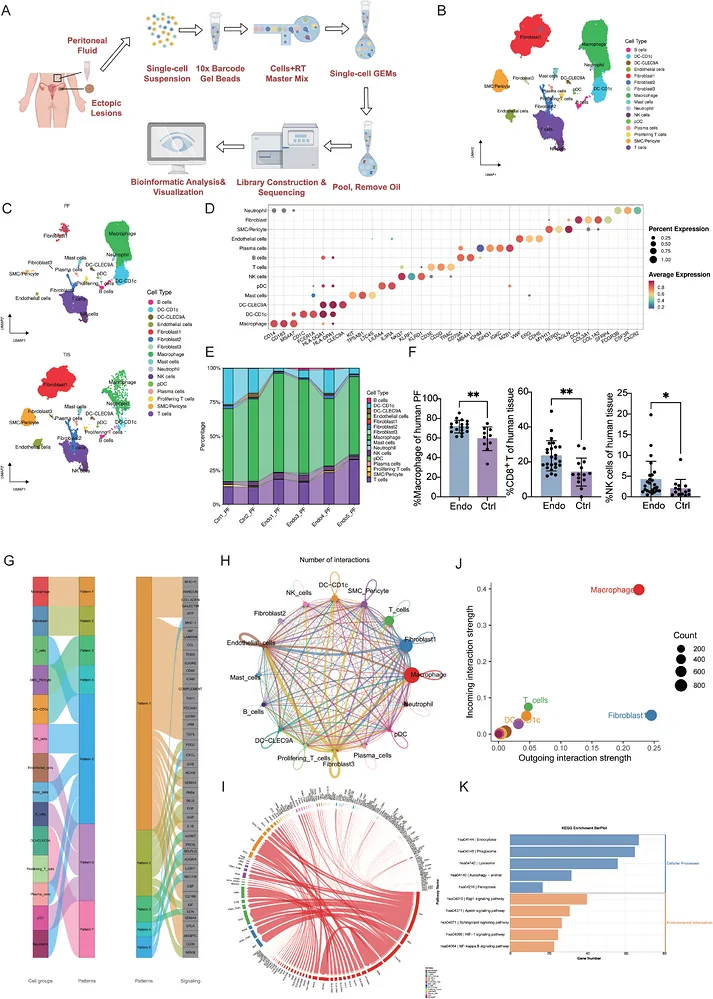
Single‐cell transcriptomic profiling of the cellular landscape in endometriosis. (A) Schematic representation of the experimental strategy. (B) UMAP visualization of 83,572 cells from 11 samples. (C) UMAP plots split by PF (top) and tissue (bottom) groups. (D) Dot plot showing canonical marker genes used to annotate each cell type. Dot size reflects the fraction of expressing cells, and color depth reflects mean expression. (E) Cell‐type composition of PF samples across individuals. (F) Flow cytometric validation of macrophage proportions in PF (left; Endo, *n* = 18; Ctrl, *n* = 10) and CD8^+^ T cell (middle) and NK cell (right) proportions in tissues (Endo, *n* = 25; Ctrl, *n* = 14). (G) Alluvial plot of intercellular signaling patterns. Cell types are connected to communication patterns and their corresponding signaling pathways. Ribbon width is proportional to the relative contribution of each cell type to a given pattern. (H) Cell–cell communication network. Node size reflects total interaction count, and edge width reflects the number of ligand–receptor pairs. (I) Chord diagram of pairwise ligand–receptor interactions. Line color denotes the sender cell type, and line width indicates interaction strength. (J) Outgoing versus incoming interaction strength for each cell type. Macrophages ranked highest on both axes. (K) KEGG enrichment of macrophage‐associated genes, ordered by gene count. Data are presented as mean ± SEM. The specific statistical tests used for each panel are as follows: (F): Unpaired two‐tailed Mann‐Whitney U test. ^*^ *p* < 0.05, ^**^ *p* < 0.01.

Cell–cell communication analysis (Figures 1G–K) identified macrophages as the most connected cell type in the intercellular network. Compared with other immune cells such as DCs, macrophages interacted most extensively with fibroblasts and T cells and involved more signaling pathways (Figure 1H), with dense ligand–receptor pairs (Figure 1I) and the strongest interaction intensity among all cell types (Figure 1J). Pathway enrichment of macrophage‐associated genes highlighted phagocytosis and ferroptosis (Figure 1K), suggesting that macrophages play a dominant role in cell–cell communication within the endometriotic microenvironment.

### 2.2 TREM2^+^ Macrophages Are Markedly Enriched in Endometriosis

As macrophages dominated the intercellular communication network, we performed unsupervised clustering of macrophages and resolved 10 clusters (Figure S2A). After batch‐effect correction, clusters 1, 2, 6, 7, and 8 were present in both tissue and PF samples (Figure 2A) and were retained for subsequent analysis. These five clusters were annotated as resident‐like, M1‐like, M2‐like, APOE^+^, and TREM2^+^ macrophages. Functional enrichment analysis of the top 50 marker genes highlighted a specific transcriptional profile of the TREM2^+^ subset (Figure 2B). TREM2^+^ macrophages co‐expressed TREM2 and CD63, distinguishing them from M1‐like and M2‐like macrophages (Figure 2C). M1‐like macrophages expressed inflammatory cytokine genes such as IL1B, CXCL2, CXCL3, and CXCL8, whereas M2‐like macrophages expressed MRC1 and MAF at high levels (Figure S2B,C). RNA velocity analysis suggested that all subsets diverged from a resident‐like macrophage state along relatively independent trajectories (Figure 2D).

**FIGURE 2:**
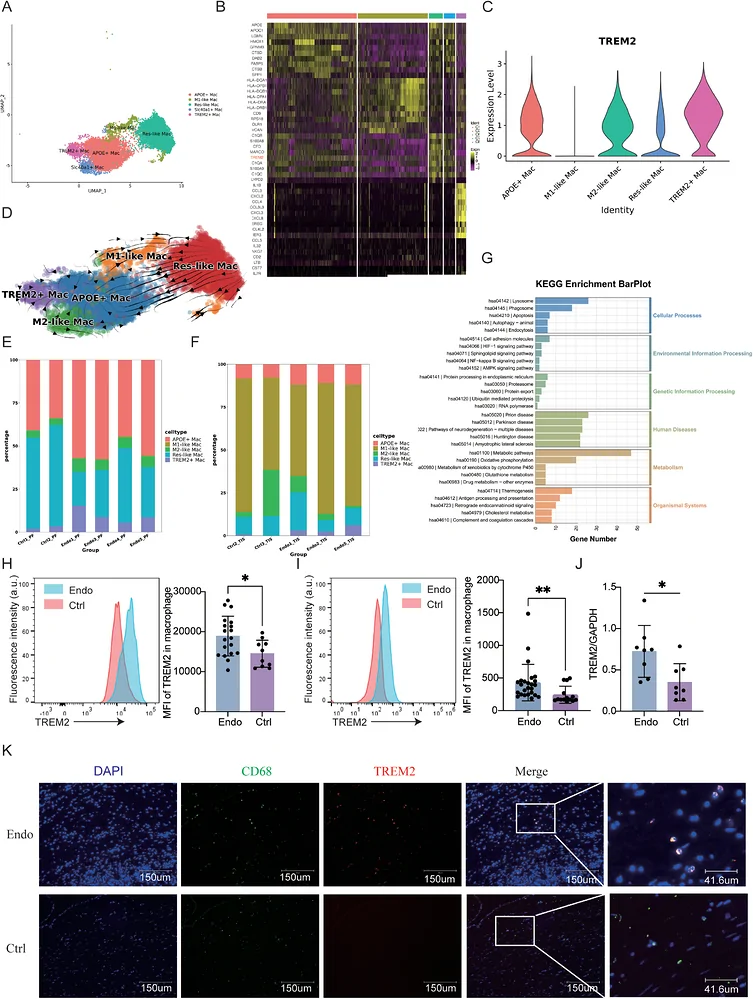
TREM2+ macrophages are markedly enriched in endometriosis. (A) UMAP plot of macrophage subclusters shared across PF and tissue samples. (B) Heatmap of differentially expressed genes across the five macrophage subpopulations. (C) Violin plot of TREM2 expression across macrophage subsets. (D) RNA velocity streamlines projected onto the UMAP embedding. (E, F) Stacked bar charts of macrophage subset composition in PF (E) and tissue (F) samples from individual patients. (G) KEGG pathway enrichment of the top 100 marker genes of TREM2^+^ macrophages, grouped by pathway category. (H) Representative flow cytometry image and quantification of TREM2 MFI in PF macrophages (Endo, *n* = 18; Ctrl, *n* = 10). (I) Representative flow cytometry image and quantification of TREM2 MFI in tissue macrophages (Endo, n = 25; Ctrl, *n* = 14). (J) Western blot quantification of TREM2 protein levels in PF macrophages (*n* = 8 per group). (K) Representative immunofluorescence images of ectopic lesions and control cysts. CD68 (green), TREM2 (red), DAPI (blue). Boxed regions are magnified on the right. Scale bars: 150 µm (left four columns) and 41.6 µm (magnified panels). Data are presented as mean ± SEM. The specific statistical tests used for each panel are as follows: (H, I): Unpaired two‐tailed Mann‐Whitney U test. (J): Unpaired two‐tailed Student's *t*‐test. ^*^ *p* < 0.05, ^**^ *p* < 0.01.

Comparison of macrophage subpopulation distributions showed that TREM2^+^ macrophages were proportionally increased in patients with endometriosis across both tissue and PF samples (Figure 2E,F). KEGG pathway analysis of the top 100 marker genes of the TREM2^+^ subset revealed enrichment in lysosome, phagosome, metabolic, and oxidative phosphorylation pathways, as well as antigen presentation and T cell activation pathways, suggesting crosstalk with T cells (Figure 2G).

Flow cytometry confirmed higher TREM2 expression in macrophages from both PF and tissues of patients with endometriosis compared with controls (Figure 2H,I). TREM2 protein levels were also significantly elevated in endometriotic samples (Figure 2J), and immunofluorescence showed co‐localization of TREM2 with CD68 in endometriotic lesions (Figure 2K). M2‐associated markers were concurrently increased (Figure S2D). TREM2^+^ and M2‐like macrophages were co‐enriched in endometriosis, and the functional relationship between the two subsets remains to be determined. Reference mapping of a published TREM2^+^ macrophage gene signature onto our dataset showed significant overlap with the endometriosis‐associated macrophage cluster (Figure S2E) [22], supporting the similarity of this cluster to previously described TREM2^+^ macrophages. These results indicate that TREM2^+^ macrophages are enriched in endometriosis and may influence the local immune microenvironment.

### 2.3 CD8^+^ T Cells in Endometriosis Exhibit a Functionally Exhausted Phenotype

We next examined CD8^+^ T and NK cells to determine whether effector lymphocytes were functionally altered in endometriosis. Reclustering of the T cell population revealed significant sample‐type specificity. Based on canonical marker genes, we annotated the clusters as naive CD8^+^, CD4^+^, mucosal‐associated invariant T (MAIT), regulatory T (Treg), γδ T, PF‐associated CD8^+^ T cells, and tissue‐associated CD8^+^ T cells (Figure 3A,B). In the PF compartment, CD8^+^ T cells from patients with endometriosis showed higher CD69 expression than those from controls (Figure 3C). CD69 is an early T cell activation marker and may also be associated with persistent activation under chronic stimulation [23]. Consistent with a dysfunctional state, T Cell State Identifier analysis showed elevated terminal exhaustion scores in PF CD8^+^ T cells from patients with endometriosis (Figure 3D). Flow cytometric validation with an expanded cohort confirmed that these cells had reduced perforin expression, indicating reduced cytotoxicity (Figure 3E).

**FIGURE 3:**
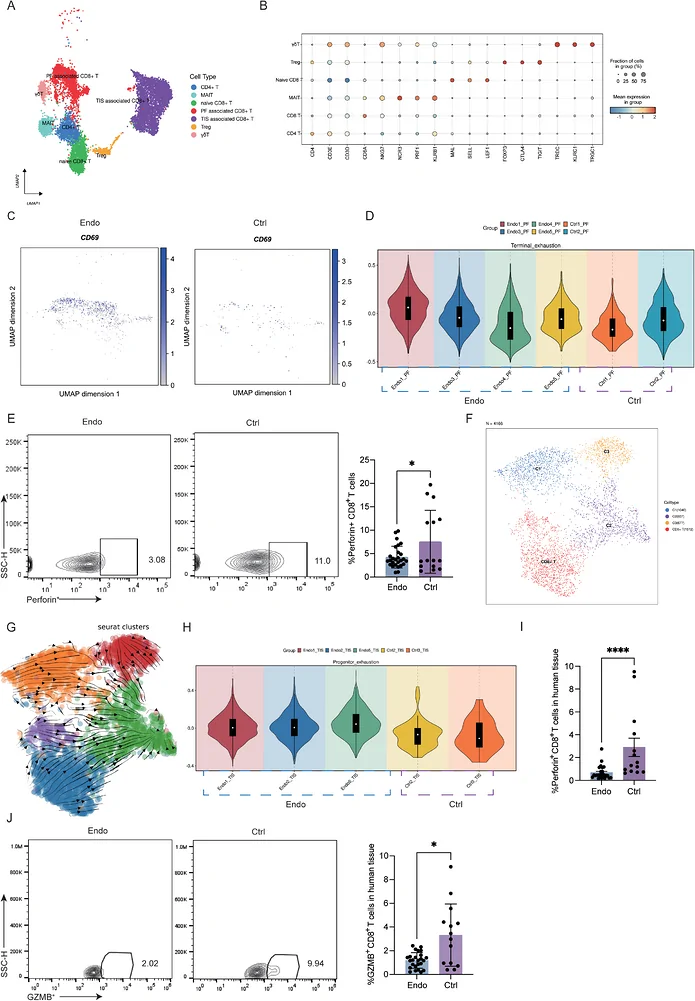
CD8^+^ T cells in endometriosis exhibit a functionally exhausted phenotype. (A) UMAP visualization of T cell subclusters across all samples. (B) Dot plot of canonical marker genes used for T cell subset annotation. (C) Feature plots of CD69 expression on the CD8^+^ T cell UMAP. (D) Violin plot of terminal exhaustion signature scores in PF CD8^+^ T cells, shown per sample. Endo samples (left) and Ctrl samples (right) are separated by the dashed line. (E) Representative flow cytometry images and quantification of perforin^+^ CD8^+^ T cell percentage in PF (Endo, *n* = 27; Ctrl, *n* = 15). (F) UMAP of re‐clustered CD8^+^ T cells from tissue samples. (G) RNA velocity streamlines projected onto the tissue CD8^+^ T cell UMAP, colored by Seurat cluster identity. (H) Violin plots of progenitor exhaustion signature scores in tissue‐derived CD8^+^ T cells. (I) Quantification of perforin^+^ CD8^+^ T cell percentage in tissues (Endo, *n* = 25; Ctrl, *n* = 14). (J) Representative flow cytometry images and quantification of GZMB^+^ CD8^+^ T cell percentage in tissues (Endo, *n* = 25; Ctrl, *n* = 14). Data are presented as mean ± SEM. The specific statistical tests used for each panel are as follows: (E): Unpaired two‐tailed Student's *t*‐test; (I, J): Unpaired two‐tailed Mann‐Whitney U test. ^*^ *p* < 0.05, ^****^ *p* < 0.0001.

We then isolated and sub‐clustered tissue‐associated CD8^+^ T cells (Figures 3F and S3A). This population was expanded in ectopic lesions compared with control tissues. RNA velocity analysis suggested that Cluster 2 represented an upstream state along the inferred trajectory, with subsequent transitions toward other CD8^+^ T cell states (Figure 3G). Progenitor exhaustion scores further supported this trajectory (Figure 3H), and flow cytometric analysis of additional clinical samples showed decreased GZMB and perforin expression in tissue CD8^+^ T cells from patients with endometriosis (Figure 3I,J). These findings indicate that CD8^+^ T cells in both PF and ectopic tissues of patients with endometriosis exhibit features of functional exhaustion accompanied by reduced cytotoxic molecule expression.

### 2.4 Impaired Functions of NK Cells Within the Endometriosis Immune Landscape

Given that NK cells represent another major cytotoxic population in PF, we next examined NK cell subsets within the endometriotic microenvironment. Unsupervised clustering identified PF‐associated and tissue‐associated NK cell populations alongside several additional clusters (Figure 4A). The proportion of CD16^+^ NK cells did not differ significantly between the endometriosis and control groups in either PF or ectopic tissues (Figure 4B). However, the inhibitory receptor KLRG1 was predominantly expressed in tissue‐associated NK cells (Figure 4C), suggesting phenotypic differences despite similar subset composition.

**FIGURE 4:**
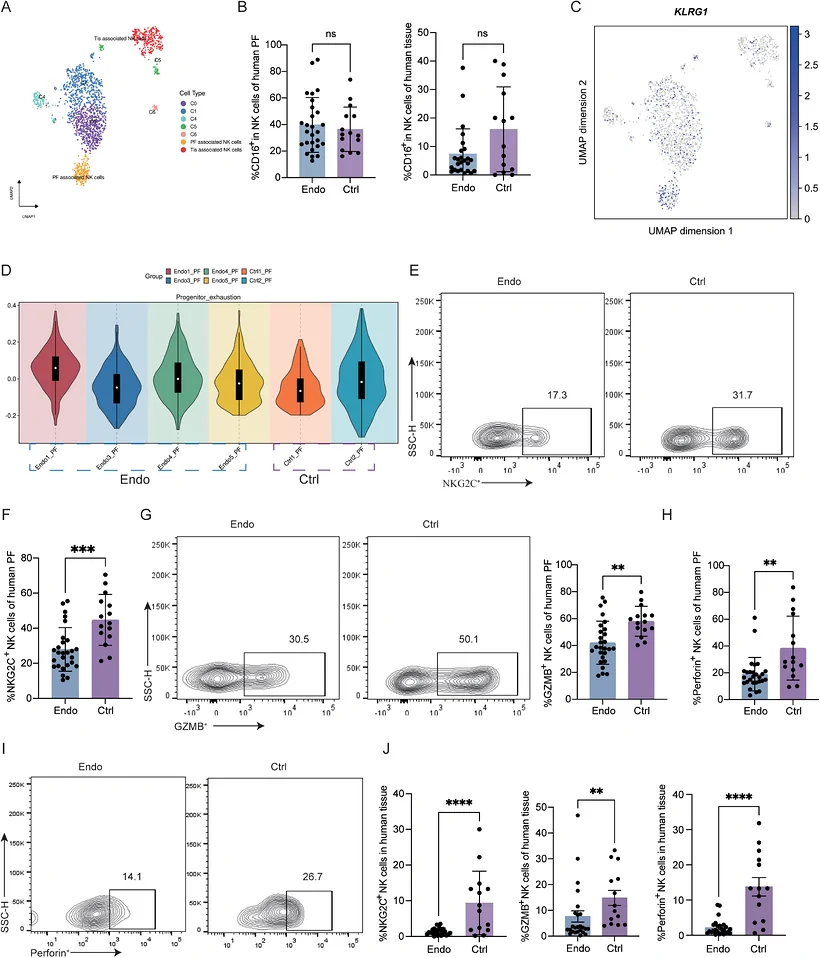
Diverse phenotypes and functions of NK cells within the endometriosis immune landscape. (A) UMAP of NK cell subclusters across all samples. (B) Percentages of CD16^+^ NK cells in PF (left, Endo, *n* = 27; Ctrl, *n* = 15) and tissue (right, Endo, *n* = 25; Ctrl, *n* = 14). (C) Feature plot of KLRG1 expression on the NK cell UMAP. (D) Violin plots of progenitor exhaustion signature scores in PF NK cells. (E) Representative flow cytometry images of NKG2C expression in PF NK cells from Endo and Ctrl groups. (F) Quantification of the NKG2C^+^ NK cell percentage in PF (Endo, *n* = 27; Ctrl, *n* = 15). (G) Representative flow cytometry images and quantification of GZMB^+^ NK cell percentage in PF (Endo, *n* = 27; Ctrl, *n* = 15). (H, I) Representative flow cytometry images and quantification of perforin^+^ NK cell percentage in PF (Endo, *n* = 27; Ctrl, *n* = 15). (J) Quantification of NKG2C^+^, GZMB^+^, and perforin^+^NK cell percentages in tissues (Endo, *n* = 25; Ctrl, *n* = 14). Data are presented as mean ± SEM. The specific statistical tests used for each panel are as follows: (F, G): Unpaired two‐tailed Student's *t*‐test; (B, H, J): Unpaired two‐tailed Mann‐Whitney U test. ^**^ *p* < 0.01, ^****^ *p* < 0.0001; ns, not significant.

As with the exhaustion phenotype observed in CD8^+^ T cells, NK cells in the PF of patients with endometriosis displayed higher progenitor exhaustion scores (Figures 4D and S3B). Flow cytometric validation with an expanded cohort confirmed that the proportions of NKG2C^+^, GZMB^+^, and Perforin^+^ NK cells were all significantly decreased in the PF of patients with endometriosis compared with controls (Figure 4E–I). NK cells from ectopic tissues showed a similar decrease in NKG2C^+^, GZMB^+^, and Perforin^+^ subsets (Figure 4J). NK cells in endometriosis exhibit exhaustion‐associated features, including reduced cytotoxicity and altered receptor expression.

### 2.5 TREM2^+^ Macrophages Regulate CD8^+^ T Cell Function Through SPP1‐CD44 Mediated Communication

We used CellPhoneDB to map ligand–receptor interactions among immune cell types in endometriosis. Macrophages exhibited the strongest interactions with other immune cell types in both control and endometriosis conditions (Figure 5A,B). Given that TREM2^+^ macrophages were enriched in endometriosis and displayed unique immunoregulatory characteristics, we further investigated whether TREM2^+^ macrophages influence cytotoxic lymphocyte function. To assess the functional effect of TREM2^+^ macrophages on CD8^+^ T cells, peritoneal macrophages isolated from wild‐type (WT) and TREM2‐deficient (KO) mice were co‐cultured with CD8^+^ T cells. Compared with WT macrophages, CD8^+^ T cells co‐cultured with KO macrophages exhibited significantly increased proliferation (Figure 5C) and decreased Pdcd1 expression at both the mRNA and protein levels (Figure 5D,E), indicating that TREM2^+^ macrophages contribute to CD8^+^ T cell dysfunction. In addition, co‐culture with KO macrophages significantly reduced the expression of the inhibitory receptor NKG2A on NK cells compared with WT macrophages (Figure S4A), suggesting that TREM2^+^ macrophages are associated with an inhibitory NK cell phenotype.

**FIGURE 5:**
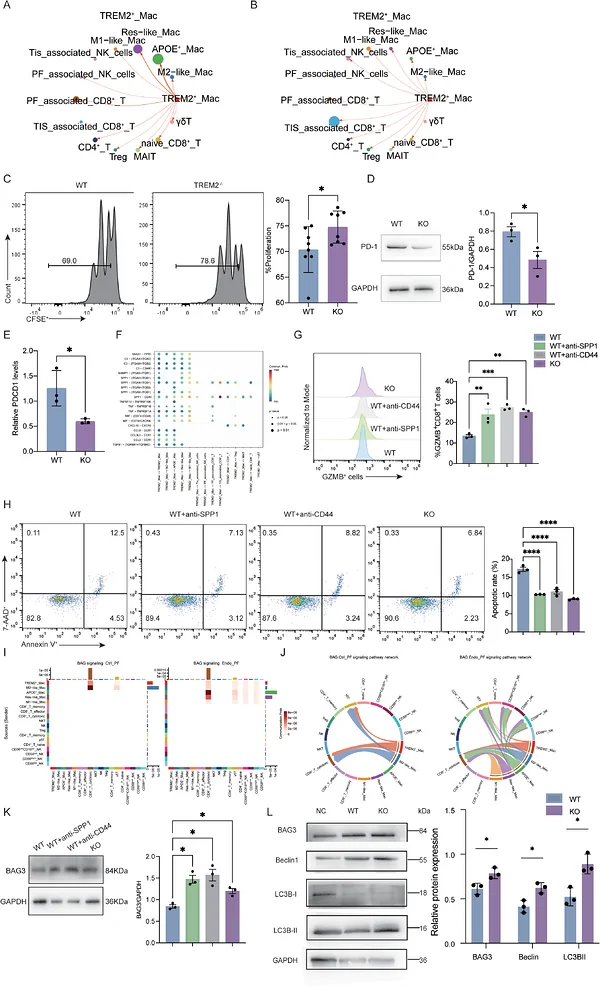
TREM2^+^ macrophages regulate CD8^+^ T cell function through SPP1‐CD44 mediated communication. (A) Cell–cell communication network depicting interactions between TREM2^+^ macrophages and other immune cell subsets in PF. (B) Cell–cell communication network depicting interactions between TREM2^+^ macrophages and other immune cell subsets in tissues. (C) Representative CFSE image and quantification of proliferation of CD8^+^ T cells co‐cultured with WT or TREM2‐KO macrophages (*n* = 8 per group). (D) Relative protein expression of PD‐1 in CD8^+^ T cells after co‐culture with WT or TREM2‐KO macrophages (*n* = 3 per group). (E) Relative Pdcd1 mRNA expression in CD8^+^ T cells after co‐culture with WT or TREM2‐KO macrophages (*n* = 3 per group). (F) Dot plot of statistically significant ligand‐ receptor interactions between TREM2^+^ macrophages and CD8^+^ T cells or NK cells in tissues. (G) Representative flow cytometry image and quantification of GZMB^+^ CD8^+^ T cell percentage after co‐culture with WT or TREM2‐KO macrophages pretreated with SPP1 neutralizing antibody or CD44 blocking antibody (*n* = 3 per group). (H) Representative flow cytometry images and quantification of apoptosis in CD8^+^ T cells after co‐culture with WT or TREM2‐KO macrophages pretreated with SPP1 neutralizing antibody or CD44 blocking antibody (*n* = 3 per group). (I) Heatmaps of BAG signaling pathway activity in cell–cell communication networks from Ctrl PF and Endo PF. (J) Chord diagrams illustrating BAG signaling pathway interactions among immune cell subsets in Ctrl PF and Endo PF. (K) Western blot analysis and quantification of BAG3 expression in CD8^+^ T cells co‐cultured with WT or TREM2‐KO macrophages pretreated with SPP1 neutralizing antibody or CD44 blocking antibody (*n* = 3 per group). (L) Representative western blots and quantification of autophagy‐related proteins in CD8^+^ T cells co‐cultured with WT or TREM2‐KO macrophages (*n* = 3 per group). Data are presented as mean ± SEM. The specific statistical tests used for each panel are as follows: (C): Unpaired two‐tailed Mann‐Whitney U test; (D, E, L): Unpaired two‐tailed Student's *t*‐test; (G, H, K): One‐way ANOVA with Tukey's multiple comparisons post‐hoc test. ^*^ *p* < 0.05, ^**^ *p* < 0.01, ^***^ *p* < 0.001, ^****^ *p* < 0.0001.

CellPhoneDB analysis identified several candidate ligand‐receptor pairs, among which SPP1‐CD44 emerged as a potential interaction between TREM2^+^ macrophages and CD8^+^ T cells (Figure 5F). To determine whether this predicted interaction contributes to CD8^+^ T cell dysfunction, we performed functional blockade experiments targeting SPP1 and CD44. Neutralization of SPP1 or CD44 significantly increased GZMB expression and decreased apoptosis of CD8^+^ T cells (Figure 5G,H), supporting the functional relevance of SPP1‐CD44 signaling in CD8^+^ T cell regulation.

After identifying the functional relevance of the SPP1‐CD44 interaction, we further investigated the molecular changes associated with this signaling pathway. Communication analysis revealed that BAG signaling among immune cell populations was reduced in endometriosis PF compared with control PF (Figure 5I–J). We examined whether SPP1‐CD44 signaling affected BAG3 expression in CD8^+^ T cells. Neutralization of SPP1 or CD44 significantly increased BAG3 expression compared with the control group (Figure 5K), suggesting that BAG3 expression is associated with SPP1‐CD44 signaling in CD8^+^ T cells. BAG3 has been implicated in the regulation of intracellular protein homeostasis and autophagy‐related processes [24]. We therefore hypothesized that changes in BAG3‐associated signaling may contribute to CD8^+^ T cell dysfunction by affecting autophagy‐related processes. Consistent with this hypothesis, western blotting and qPCR analyses showed that BAG3, Beclin1, and LC3B‐II levels were all significantly lower in CD8^+^ T cells co‐cultured with WT macrophages than in those co‐cultured with KO macrophages (Figures 5L and S4B). These results suggest that TREM2^+^ macrophages regulate CD8^+^ T cell function through SPP1‐CD44‐associated communication, accompanied by alterations in BAG3‐associated autophagy pathways.

### 2.6 BAG3 Associated Autophagy Regulation Contributes to TREM2^+^ Macrophage Induced CD8^+^ T Cell Dysfunction

We next investigated whether BAG3‐associated autophagy regulation is involved in the functional changes of CD8^+^ T cells induced by TREM2^+^ macrophages. TREM2 was silenced by siRNA in BMDMs, and knockdown efficiency was confirmed by western blot and qPCR (Figure S5A,B). Because changes in LC3B expression alone cannot distinguish altered autophagosome formation from impaired autophagic degradation, we first examined autophagic flux using Bafilomycin A1 (BafA1) treatment. Compared with CD8^+^ T cells co‐cultured with control macrophages, those exposed to siTREM2 macrophages showed reduced basal p62 expression. Following BafA1 treatment, both LC3BII and p62 accumulated in CD8^+^ T cells co‐cultured with siTREM2 macrophages (Figure 6A,B), supporting enhanced autophagic flux upon TREM2 depletion. Consistently, these cells also displayed improved mitochondrial function, characterized by increased mitochondrial membrane potential, reduced mitochondrial superoxide levels, and elevated ATP levels (Figure 6C–F). These changes were partially attenuated following BafA1 treatment, suggesting that autophagy regulation contributes to the metabolic improvement of CD8^+^ T cells. We next examined whether these molecular alterations affected CD8^+^ T cell function. Compared with the siNC group, CD8^+^ T cells co‐cultured with siTREM2 macrophages showed reduced apoptosis and elevated GZMB expression (Figure 6G–I). These functional improvements were partially reversed following BafA1 treatment, indicating that autophagy contributes to the functional recovery of CD8^+^ T cells.

**FIGURE 6:**
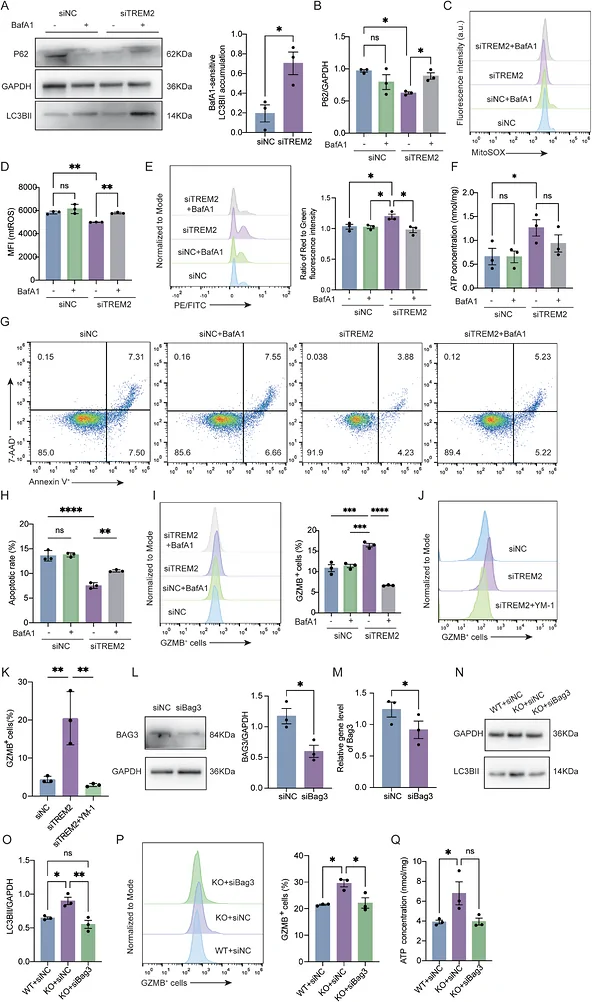
BAG3‐associated autophagy regulation contributes to TREM2^+^ macrophage‐induced CD8^+^ T cell dysfunction. (A) Representative Western blot and quantification of LC3BII accumulation following Bafilomycin A1 (BafA1) treatment (*n* = 3 per group). (B) Quantification of P62 protein expression by Western blot following BafA1 treatment (*n* = 3 per group). (C, D) Representative flow cytometry image and quantification of mitochondrial oxidative stress in CD8^+^ T cells co‐cultured with siNC or siTREM2 BMDMs following BafA1 treatment (*n* = 3 per group). (E) Representative flow cytometry image and quantification of mitochondrial membrane potential based on JC‐1 fluorescence ratio in CD8^+^ T cells co‐cultured with siNC or siTREM2 BMDMs following BafA1 treatment (*n* = 3 per group). (F) Measurement of ATP levels in CD8^+^ T cells after co‐culture with siNC or siTREM2 BMDMs with or without BafA1 treatment (*n* = 3 per group). (G, H) Representative flow cytometry images and quantification of apoptosis in CD8^+^ T cells after co‐culture with siNC or siTREM2 BMDMs with or without BafA1 treatment (*n* = 3 per group). (I) Representative flow cytometry image and quantification of GZMB^+^ CD8^+^ T cell percentage after co‐culture with siNC or siTREM2 BMDMs with or without BafA1 treatment (*n* = 3 per group). (J, K) Representative flow cytometry image and quantification of GZMB^+^ CD8^+^ T cell percentage co‐cultured with siNC, siTREM2, or siTREM2 + YM‐1 macrophages (*n* = 3 per group). (L) Representative western blot and quantification of BAG3 in siNC and siBag3 CD8^+^ T cells (*n* = 3 per group). (M) Relative Bag3 mRNA expression in siNC and siBag3 CD8^+^ T cells (*n* = 3 per group). (N, O) Representative western blot and quantification of LC3BII expression in CD8^+^ T cells transfected with siNC or siBAG3 and co‐cultured with WT or TREM2^−^/^−^ BMDMs (*n* = 3 per group). (P) Representative flow cytometry image and quantification of GZMB^+^ CD8^+^ T cell percentage after BAG3 knockdown (*n* = 3 per group). (Q) ATP measurement in CD8^+^ T cells after BAG3 knockdown (*n* = 3 per group). Data are presented as mean ± SEM. The specific statistical tests used for each panel are as follows: (A, D, F, H, I, O, P): One‐way ANOVA with Tukey's multiple comparisons post‐hoc test. (B, E, K, Q): Kruskal‐Wallis test with Dunn's multiple comparisons post‐hoc test. (L, M): Unpaired two‐tailed Student's *t*‐test. ^*^ *p* < 0.05, ^**^ *p* < 0.01, ^****^ *p* < 0.0001, ns, not significant.

To further investigate the involvement of BAG3‐related signaling, we disrupted the BAG3‐HSP70 interaction using YM‐1, a rhodamine‐derived allosteric inhibitor of HSP70. YM‐1 treatment attenuated the autophagy‐related changes observed in CD8^+^ T cells cultured with siTREM2 macrophages and reduced the functional recovery under these conditions (Figures 6J,K and S5C–G). These findings suggest that BAG3‐associated signaling is involved in the regulation of CD8^+^ T cell function. To further assess the functional contribution of BAG3, BAG3 was silenced in CD8^+^ T cells. BAG3 knockdown efficiency was confirmed by western blotting and qPCR (Figure 6L,M). BAG3 depletion reduced LC3B‐II expression and decreased GZMB expression in CD8^+^ T cells, with a trend toward reduced ATP production (Figure 6N–Q), indicating that BAG3 contributes to autophagy‐related processes and CD8^+^ T cell effector function.

To test the relevance of BAG3‐associated regulation in vivo, endometriosis model mice were treated with YM‐1 alone or in combination with an anti‐CD8 depleting antibody. YM‐1 administration significantly increased ectopic lesion size and weight compared with isotype controls. Co‐administration of anti‐CD8 antibody with YM‐1 did not further aggravate lesion burden (Figure S5H). The proportion of GZMB^+^ cells in peritoneal fluid was also significantly reduced in YM‐1‐treated mice (Figure S5I). These results suggest that BAG3‐associated regulation of CD8^+^ T cell function may contribute to immune dysfunction during endometriosis progression.

### 2.7 TREM2 Deficiency Enhances CD8^+^ T and NK Cell Cytotoxicity and Inhibits Endometriotic Lesion Growth

To evaluate the functional contribution of TREM2 in vivo, we established endometriosis models in WT and TREM2^−^/^−^ (KO) mice and measured ectopic lesion weight 14 days after intraperitoneal injection of endometrial fragments. Lesions in KO mice were significantly smaller than those in WT, indicating that TREM2 promotes endometriosis progression (Figure 7A,B). To assess the contribution of cytotoxic lymphocytes to the reduced lesion burden in KO mice, we depleted CD8^+^ T cells, NK cells, or both CD8^+^ T cells and NK cells. Depletion of either CD8^+^ T cells or NK cells partially reversed the protective effect of TREM2 deficiency. Combined depletion of CD8^+^ T and NK cells resulted in greater lesion burden than depletion of either population alone, suggesting that both populations contribute to lesion control in the absence of TREM2.

**FIGURE 7:**
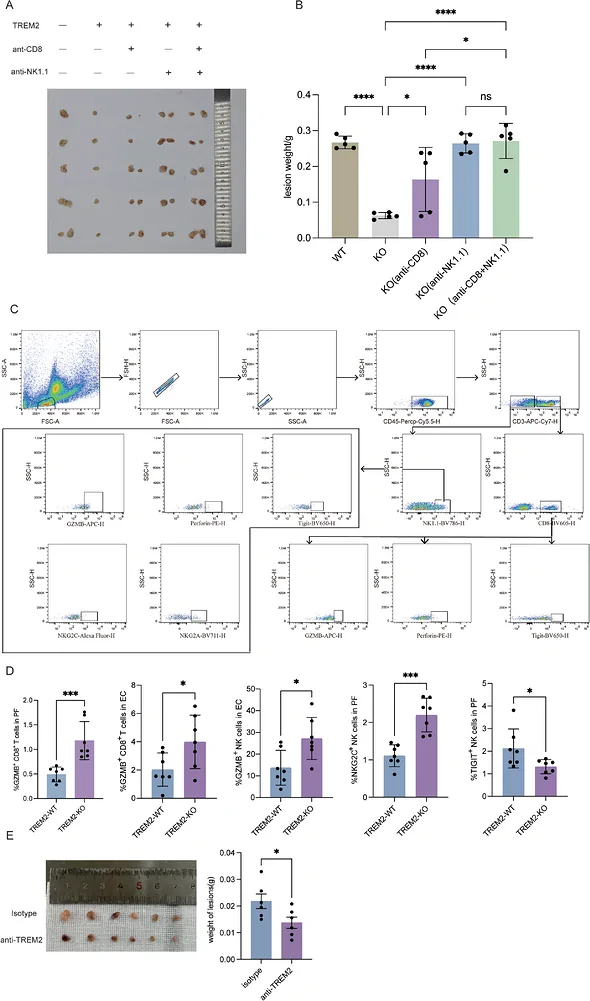
TREM2 deficiency enhances CD8^+^ T and NK cell cytotoxicity and inhibits endometriotic lesion growth. (A) Representative endometriotic lesion images in WT, TREM2‐KO, TREM2‐KO + anti‐CD8, TREM2‐KO + anti‐NK1.1, and TREM2‐KO + anti‐CD8 + anti‐NK1.1 mice. (B) Quantification of lesion weight across the five groups (*n* = 5 per group). (C) Representative flow cytometry gating strategy for analyzing functional markers of CD8^+^ T cells and NK cells in PF and ectopic lesions from WT and TREM2‐KO mice. (D) Quantification of functional markers by flow cytometry in WT and TREM2‐KO mice (*n* = 7 per group). (E) Representative endometriotic lesion image and quantification of lesion weight between isotype and anti‐TREM2 group (*n* = 6 per group). Data are presented as mean ± SEM. The specific statistical tests used for each panel are as follows: (B): One‐way ANOVA with Tukey's multiple comparisons post‐hoc test. (D, E): Unpaired two‐tailed Student's *t*‐test. ^*^ *p* < 0.05, ^***^ *p* < 0.001, ^****^ *p* < 0.0001, ns, not significant.

Flow cytometric profiling of PF and ectopic lesions revealed a clear shift in effector cell phenotype in KO mice (Figure 7C). The proportion of GZMB^+^ CD8^+^ T cells was significantly higher in both PF and ectopic lesions of KO mice compared with WT controls (Figure 7D), consistent with enhanced CD8^+^ T cell cytotoxic activity following TREM2 deficiency. A similar increase in GZMB^+^ NK cells was observed in PF. Beyond cytotoxicity markers, peritoneal NK cells in KO mice displayed upregulated expression of the activating receptor NKG2C and decreased expression of the inhibitory checkpoint T‐cell immunoreceptor with Ig and ITIM domains (TIGIT). These results indicate that TREM2 deficiency enhances CD8^+^ T and NK cell cytotoxicity and inhibits ectopic lesion growth.

To evaluate the therapeutic potential of targeting TREM2, WT mice with endometriosis were treated with a TREM2 neutralizing antibody. Pharmacological blockade of TREM2 significantly reduced ectopic lesion size and weight compared with control antibody treatment (Figure 7E). These findings may provide a potential non‐hormonal therapeutic target for endometriosis.

## 3 Discussion

In this study, we used single‐cell transcriptomics and functional experiments to characterize the immune microenvironment in endometriosis. We identified a transcriptionally distinct TREM2^+^ macrophage population enriched in endometriotic tissue and peritoneal fluid that is associated with impaired CD8^+^ T cell and NK cell cytotoxicity. Mechanistically, our findings suggest that TREM2^+^ macrophages regulate CD8^+^ T cell function through SPP1‐CD44‐mediated communication and BAG3‐associated autophagy regulation. These findings identify TREM2^+^ macrophages as an important immunoregulatory population in endometriosis and provide a rationale for further investigating TREM2 as a potential non‐hormonal therapeutic target.

Previous single‐cell studies have revealed substantial immune‐cell heterogeneity in endometriosis, including macrophage states and immune dysfunction in the peritoneal microenvironment [25, 26]. Our design instead incorporated PF and ectopic tissue samples and focused on the interaction between macrophage subsets and cytotoxic lymphocytes. This strategy identified macrophages as the most connected cell type in the intercellular communication network. TREM2^+^ macrophages were significantly enriched in both ectopic lesions and PF, which we confirmed in clinical samples. TREM2 has been implicated in immunosuppressive macrophage states in several malignancies, where TREM2^+^ macrophages are associated with impaired T cell responses, while genetic or pharmacological targeting of TREM2 can remodel the tumor myeloid compartment, enhance antitumor immunity, and improve responses to immune checkpoint blockade [27, 28, 29, 30, 31]. These findings are consistent with our observation that TREM2 knockout reduced endometriotic lesion burden in vivo. Within macrophages, RNA velocity analysis suggested that all subsets diverged from a resident‐like macrophage state along relatively independent trajectories. KEGG/GO enrichment pointed to lysosome, phagosome, and oxidative phosphorylation pathways more than typical inflammatory or tissue‐repair signatures, highlighting the limitations of classifying macrophage states solely according to the classical M1/M2 framework [32, 33]. This metabolic profile is reminiscent of tumor‐associated macrophage states in which metabolic reprogramming contributes to immunosuppressive and pro‐disease functions [34, 35], suggesting that TREM2^+^ macrophages may adopt a comparable regulatory phenotype in endometriosis.

Our single‐cell, flow cytometric, and co‐culture data show that TREM2^+^ macrophages promote a dysfunctional phenotype in both CD8^+^ T cells and NK cells in the endometriotic microenvironment. The CD8^+^ T cell dysfunction observed in our study shares features with T cell exhaustion described under chronic stimulation and in tumor microenvironments [36, 37, 38]. NK cells also showed elevated progenitor exhaustion scores, consistent with dysfunctional NK cell states described in tumor microenvironments [39, 40, 41]. Our co‐culture and knockout experiments further support an important role for TREM2 in regulating the suppressive phenotype of cytotoxic lymphocytes. Co‐culture with peritoneal macrophages from TREM2^−^/^−^ mice resulted in lower PD‐1 expression on CD8^+^ T cells compared with WT macrophages. In vivo, TREM2 deletion inhibited lesion growth, and depleting either CD8^+^ T cells or NK cells partially reversed this effect. Combined depletion further increased lesion burden beyond that seen with single depletion, indicating that the two effector populations contribute to lesion control. These findings suggest that immune dysfunction in endometriosis is not simply a consequence of the hormonal environment but involves active regulation of immune cell interactions [42, 43] in which TREM2^+^ macrophages impair effector lymphocyte function.

Mechanistically, our data indicate that TREM2^+^ macrophages regulate CD8^+^ T cell function through alterations in BAG3‐associated autophagy pathways. Our CellPhoneDB analysis identified the SPP1‐CD44 interaction as a candidate communication pathway between TREM2^+^ macrophages and CD8^+^ T cells, and functional blockade of SPP1 or CD44 partially restored CD8^+^ T cell activity, suggesting a potential link between TREM2^+^ macrophages and CD8^+^ T cell regulation [44]. BAG3 is an HSP70 co‐chaperone involved in chaperone‐assisted selective autophagy and the autophagic clearance of damaged or misfolded proteins [24, 45]. Autophagy, in turn, has an important role in CD8^+^ T cell survival, metabolic regulation, and effector function [46, 47]. Consistent with these observations, CD8^+^ T cells exposed to TREM2‐depleted macrophages exhibited increased BAG3 expression, enhanced autophagy‐related markers, improved cellular metabolic status, and enhanced effector activity. Conversely, disruption of BAG3‐HSP70 interaction using YM‐1 attenuated these functional improvements. In addition, BAG3 knockdown directly reduced LC3BII expression, GZMB production, and ATP levels in CD8^+^ T cells, further supporting the involvement of BAG3‐associated autophagy regulation in maintaining CD8^+^ T cell function. In vivo, YM‐1 treatment increased lesion burden, and this effect was not further enhanced by CD8^+^ T cell depletion, suggesting that the effects of pharmacological disruption of this interaction are at least partly associated with altered CD8^+^ T cell activity. However, the contribution of other immune populations cannot be excluded.

The translational implications of targeting TREM2 warrant further consideration. Current therapeutic strategies for endometriosis largely depend on hormonal modulation, and approaches that target disease‐associated immune dysfunction may provide complementary non‐hormonal options [48]. The enrichment of TREM2^+^ macrophages in endometriotic lesions and PF, together with the protective effects observed following genetic deletion and antibody blockade of TREM2 in mice, supports the potential of TREM2 as an immunotherapeutic target. However, the physiological functions of TREM2 in myeloid cell homeostasis and immune regulation must be considered before clinical application [49]. Systemic TREM2 inhibition may influence immune processes beyond the disease context, and its effects on fertility, endometrial receptivity, implantation, and pregnancy maintenance require careful evaluation. Likewise, although BAG3‐associated pathways regulate CD8^+^ T cell function in our model, the consequences of modulating BAG3 activity in reproductive tissues remain unknown [50]. Further studies using appropriate reproductive models and long‐term safety assessments will be needed to evaluate the feasibility of targeting TREM2‐associated immune regulation for endometriosis treatment.

Several limitations should be acknowledged. First, although our single‐cell analysis provided insights into macrophage heterogeneity in endometriosis, the control cohort included only three individuals. This limited sample size may affect the statistical power and generalizability of comparisons between endometriosis and non‐endometriosis conditions. Validation in larger, independent cohorts with balanced case‐control designs will be necessary to confirm the functional relevance of TREM2^+^ macrophages. Second, although our BAG3 knockdown and pharmacological inhibition experiments support the involvement of BAG3‐associated regulation in CD8^+^ T cell dysfunction, the upstream signaling linking TREM2^+^ macrophages to BAG3 alteration in CD8^+^ T cells remains incompletely defined. Further studies, including approaches to restore BAG3 activity, will be required to further define the causal contribution of TREM2^+^ macrophage‐derived signals to BAG3‐associated CD8^+^ T cell regulation. Thirdly, although TREM2^+^ macrophages influenced NK cell activation status and NK cell depletion partially reversed the protective effect of TREM2 deficiency, the molecular mechanisms underlying macrophage‐mediated NK cell regulation were not fully characterized. Finally, although TREM2 blockade showed therapeutic potential in mice, validation in human cohorts and further translational studies are required before clinical application.

## 4 Conclusion

In summary, our work identifies TREM2^+^ macrophages as an important immunoregulatory population in endometriosis that contributes to impaired CD8^+^ T cell and NK cell function. TREM2^+^ macrophages regulate CD8^+^ T cell activity through SPP1‐CD44‐associated communication and BAG3‐associated autophagy regulation. These findings provide new insights into macrophage‐mediated immune dysfunction in endometriosis and highlight TREM2 as a potential non‐hormonal therapeutic target for further investigation.

## 5 Materials and Methods

### 5.1 Ethics Statement

Mice were handled in accordance with the Guide for the Care of Laboratory Animals, and all procedures were approved by the Ethics Committee of Medical Integration and Practice Center of Shandong University (SDULCLL2022‐2‐17; approval date: 4 November 2022).

All human experiments were approved by the Ethics Committee of Medical Integration and Practice Center of Shandong University (SDULCLL2022‐1‐21; approval date: 4 November 2022). Written informed consent was obtained from each participant prior to sample collection.

### 5.2 Patients and Sample Collection

All clinical tissue specimens were collected from patients at the Department of Obstetrics and Gynecology, Shandong Provincial Hospital, affiliated with Shandong First Medical University. The study cohort comprised 11 samples for single‐cell RNA sequencing (scRNA‐seq) and an independent validation set of 52 endometriosis patients and 29 controls for flow cytometry analysis. The specific sample sizes for each comparison are indicated in the corresponding figure legends. Women aged 18–50 years with histopathologically confirmed ovarian endometriosis were enrolled in the endometriosis group, and controls had histopathologically confirmed benign ovarian lesions (cystic teratoma, serous cystadenoma, mucinous cystadenoma, or simple cyst). Patients with severe systemic disease, malignant tumors, pregnancy or lactation, prior pelvic or abdominal surgery, or hormonal medication use within 3 months before surgery were excluded.

Peritoneal fluid was aspirated laparoscopically, and ectopic lesions were excised. All specimens were placed in sterile Dulbecco's modified Eagle's medium/F‐12 (DMEM/F‐12; Gibco, Grand Island, NY, USA) and transferred to the laboratory within 1 h of collection. Clinicopathological information was obtained from the patients’ medical records.

### 5.3 Isolation of Single Cells From Tissues and Peritoneal Fluid

After washing with 1× PBS three times, samples were cut into fragments smaller than 1 mm^3^ and were digested in 0.125% (w/v) collagenase (LS004148, Worthington Biochemical Corp., Lakewood, NJ, USA) at 37°C for up to 1 h. The mixture was sequentially filtered through a 70 µm cell strainer and centrifuged at 300 g for 5 min. Pelleted cells were incubated in red blood cell lysis solution (R1010, Solarbio, China) to lyse red blood cells and then washed with PBS with 2% (v/v) fetal bovine serum (FBS; Gibco, Grand Island, NY, USA). Peritoneal fluid was centrifuged at 400 g for 10 min at 4°C, and cells were resuspended in PBS with 2% FBS.

Cell viability was assessed with trypan blue and confirmed to be above 85%. Single‐cell suspensions were counted on a hemocytometer and adjusted to 700–1200 cells/µL.

### 5.4 Chromium 10× Genomics Library and Sequencing

Single‐cell suspensions were loaded into a 10× Chromium Controller according to kit instructions to capture 10000 single cells, followed by cDNA amplification and library construction according to standard procedures. Sequencing was performed using an Illumina NovaSeq 6000 sequencing system (paired‐end multiplex run, 150 bp) by LC‐Bio Technology Co. Ltd. (Hangzhou, China) with a minimum depth of 20,000 reads per cell.

### 5.5 Cell Clustering Analysis, Visualization, and Annotation

Cells were clustered using the FindClusters function in Seurat, with the resolution parameter adjusted for each level of analysis. Before sub‐clustering, cells with a ribosomal gene fraction exceeding 35% were excluded. Clusters and sub‐clusters were visualized by uniform manifold approximation and projection (UMAP) and annotated on the basis of differentially expressed genes and established cell‐type markers.

### 5.6 Assessment of the Immune Functions of CD8^+^ T and NK Cell States

The functional states of CD8^+^ T cells and NK cells were assessed using TCellSI [51]. Briefly, eight marker gene sets corresponding to distinct cell states (resting, regulatory, proliferative, helper, cytotoxic, precursor exhausted, terminally exhausted, and senescent) were defined on the basis of literature curation, single‐cell data analysis, and machine‐learning approaches. For each gene in the marker sets, a preliminary score was computed using the Mann–Whitney U method by calculating the difference between its rank in the expression data and the minimum rank. Final state scores were obtained by adjusting preliminary scores with weights derived from the expression patterns of the marker gene sets and reference profiles.

### 5.7 Cell State Identification

Cell states and developmental trajectories were inferred using the CellTrails package. Highly variable genes were first identified with M3Drop, which fits a Michaelis–Menten dropout model to scRNA‐seq expression data and selects differentially expressed genes at an FDR threshold of 5%. Cell states were then determined through spectral embedding and hierarchical clustering, and clusters were visualized in a force‐directed layout based on log ratios of high‐weight paths between clusters. Marker genes for each cluster were identified using the Wilcoxon rank‐sum test at an FDR threshold of 1%.

### 5.8 Pathway Enrichment Analysis

Differentially expressed genes identified in each cluster were subjected to Gene Ontology (GO) enrichment analysis, including biological process (BP), cellular component (CC), and molecular function (MF) categories, and Kyoto Encyclopedia of Genes and Genomes (KEGG) pathway enrichment analysis using the clusterProfiler package. Enriched terms and pathways were visualized using dot plots and bar plots. Adjusted p‐values < 0.05 were considered statistically significant.

### 5.9 Cell–Cell Communication Analysis

Cell–cell communication was analyzed using CellChat and CellPhoneDB. CellChat was used to characterize global intercellular communication networks and signaling pathway‐level interactions among annotated cell populations. The expression of known ligand‐receptor pairs was used to infer communication probabilities and signaling interactions between cell types. Global communication networks, incoming and outgoing interaction strengths, communication patterns, and pathway‐specific signaling networks were subsequently analyzed and visualized using CellChat.

CellPhoneDB was used to further identify candidate ligand‐receptor interactions among macrophages, CD8^+^ T cells, and NK cells. Pairwise comparisons were performed between the indicated cell populations. Cluster labels were randomly permuted 1,000 times to determine the mean expression levels of each receptor and ligand for each interacting cell pair, thereby generating a null distribution for each receptor‐ligand pair. Interactions with *p* < 0.05 were considered significant and were visualized using dot plots and heatmaps.

### 5.10 RNA Velocity Analysis

RNA velocity was estimated using scVelo. Spliced and unspliced read counts were quantified using velocyto, and velocity vectors were computed using the dynamical model with default parameters. Velocity streamlines were projected onto the UMAP embedding to infer the directionality of cell state transitions.

### 5.11 Flow Cytometry Analysis

The cells collected from the peritoneal fluid, eutopic and ectopic tissue of both human and mouse were first stained with fluorochrome‐conjugated surface marker antibodies in the dark for 30 min at 4°C. For intracellular staining, cells were fixed and permeabilized using the BD Cytofix/Cytoperm kit (554722, BD Biosciences, USA) and washed twice with BD Perm/Wash buffer. Cells were then incubated with intracellular antibodies in the dark for 1 h at 4°C. The samples were analyzed using a ThermoFisher Attune NxT (Thermo Scientific, USA), and the data were analyzed using FlowJo 10.5.3 software (TreeStar, Ashland, OR, USA). Antibodies for staining are listed in Tables S2 and S3.

### 5.12 CD8^+^ T Cell Proliferation Assay

Spleens were harvested and mechanically dissociated into single‐cell suspensions. CD8^+^ T cells were isolated using the EasySep Mouse CD8^+^ T Cell Isolation Kit (19853, STEMCELL Technologies, USA) according to the manufacturer's instructions. Isolated CD8^+^ T cells were washed with PBS, resuspended at 15 × 10^6^/mL in PBS, and stained with CellTrace CFSE (C34570, Thermo Fisher Scientific, USA) at a final concentration of 5 µm per 1 × 10^7^ cells at 37°C for 15 min. Labeled CD8^+^ T cells were washed and resuspended at 1 × 10^6^/mL in complete RPMI 1640 medium supplemented with HEPES, L‐glutamine, 10% FBS, 1× non‐essential amino acids, 100 U/mL penicillin, 100 µg/mL streptomycin, 1 mM sodium pyruvate, and 55 µm β‐mercaptoethanol. CFSE‐labeled CD8^+^ T cells were plated at 4 × 10^4^ cells per well in U‐bottom 96‐well plates and activated with Dynabeads Mouse T‐Activator CD3/CD28 (11453D, Thermo Fisher Scientific, USA) at five times the manufacturer's recommended concentration (125 µL per 10^6^ cells). Sorted CD11b^+^F4/80^+^ macrophages were added at a macrophage‐to‐T cell ratio of 1:8 (5 × 10^3^ macrophages to 4 × 10^4^ T cells). After 3 days of co‐culture, cells were collected and stained with fluorochrome‐conjugated anti‐CD8 antibody (100743, BioLegend, USA). The samples were analyzed using a ThermoFisher Attune NxT (Thermo Scientific, USA).

### 5.13 Isolation and Culture of NK Cells

Freshly obtained mouse spleen was mechanically dissociated through 70 µm filter and resuspended in red blood cell lysis solution. Then, cells were stained for 30 min at 4°C with pre‐conjugated fluorescently labeled antibody NK1.1 (417‐5941‐82, BV786, Thermo Scientific, USA), and cells were sorted by CytoFLEX SRT (Beckman, USA). Purified and enriched NK cells were co‐cultured with sorted CD11b^+^ F4/80^+^ cells in contact (8:1) in vitro for 48 h at 37°C and then used for flow cytometry analysis.

### 5.14 Mouse Model

Wild‐type (WT) C57BL/6J mice and TREM2^−^/^−^ mice on a C57BL/6J background were purchased from Cyagen Biosciences (Suzhou, China). The endometriosis mouse model was established by intraperitoneal injection of syngeneic uterine fragments. Briefly, the estrous cycle of recipient mice was synchronized by subcutaneous injection of estradiol (3 µg in 30 µL DMSO, HY‐B0141, MCE, USA) at the nape of the neck. Uterine tissue from donor mice was minced into small fragments and injected intraperitoneally into recipient mice.

Recipient mice were divided into five groups (n = 5 per group), including WT, TREM2^−^/^−^, TREM2^−^/^−^ + anti‐CD8, TREM2^−^/^−^ + anti‐NK, and TREM2^−^/^−^ + anti‐CD8 + anti‐NK. For CD8^+^ T cell depletion, mice received intraperitoneal injections of 200 µg anti‐mouse CD8 antibody (BE0061, Bio X Cell, USA) every 3 days. For NK cell depletion, mice received intraperitoneal injections of 200 µg anti‐mouse NK1.1 antibody (BE0036, Bio X Cell, USA) every 3 days.

For pharmacological disruption of the BAG3‐HSP70 interaction, an independent cohort of WT recipient mice was divided into isotype, isotype + YM‐1, and YM‐1 + anti‐CD8 groups (*n* = 6 per group). YM‐1 (HY‐18399, MedChemExpress) was administered intraperitoneally at 2 mg/kg every other day. Anti‐mouse CD8 antibody was administered as described above. At 14 days after model establishment, ectopic lesions and peritoneal cells were collected for subsequent analysis.

To evaluate the therapeutic effect of TREM2 blockade, an independent cohort of WT recipient mice was randomly divided into isotype control and anti‐TREM2 antibody treatment groups. Mice received intraperitoneal injection of 200 µg anti‐mouse TREM2 antibody (BE0447, Bio X Cell, USA) or isotype control antibody every 5 days for a total of three administrations. At 12 days after model establishment, ectopic lesions and peritoneal cells were collected for subsequent analysis.

### 5.15 Immunofluorescence

Slides were incubated at 4°C with primary antibodies overnight, including CD68 Polyclonal antibody (25747‐1‐AP, Proteintech, China, 1:200) and TREM2 antibody (MAB17291, R&D Systems, USA, 1:200). After washes in TBST for 5 min x 3, slides were incubated at 37°C for 1 h with corresponding fluorescent secondary antibodies. Nuclei were visualized with DAPI, and images were captured using an EVOS imaging system.

### 5.16 RNA Extraction and qRT‐PCR

Total RNA was extracted from the cultured cells using TRIzol reagent (15596026, Thermo Scientific, USA) according to the manufacturer's protocol, and cDNA was synthesized using Evo M‐MLV RT Premix for qPCR (AG11706, Accurate Biotechnology, China). Quantitative PCR was carried out using a QuantStudio 5 machine (Thermo Fisher Scientific, USA). The results were normalized based on GAPDH mRNA level. Primer sequences are provided in Table S4.

### 5.17 Western Blot

Cells were lysed with RIPA lysis buffer (P0013B, Beyotime, China) supplemented with phenylmethanesulfonyl fluoride (PMSF; ST507, Beyotime, China). Protein samples were treated with SDS‐PAGE loading buffer and then separated via 12.5% SDS‐PAGE and transferred onto 0.22 µm PVDF membranes (Millipore, Germany). The primary antibodies in the experiment were GAPDH Polyclonal antibody (10494‐1‐AP, Proteintech, Chicago, USA, 1:5000), TREM2 Rabbit Polyclonal antibody (A10482, ABclonal, China, 1:1000), BAG3 Rabbit Monoclonal antibody (68076‐1‐Ig, Proteintech, Chicago, USA, 1:1000), Beclin1 Polyclonal antibody (11306‐1‐AP, Proteintech, Chicago, USA, 1:1000), LC3 Monoclonal antibody (14600‐1‐AP, Proteintech, Chicago, USA, 1:1000), and p62 Monoclonal antibody (T55546S, Abmat, USA, 1:1000). The signal of blot was visualized by chemiluminescence (ECL; ABclonal, China) and was detected using CHAMPCHEMI 910 (Beijing Sage Creation Science Co., Ltd.). Three independent experiments were performed. Relative protein levels were quantified using ImageJ software (version 1.5, Wayne Rasband, National Institutes of Health, Bethesda, MD, USA) and normalized to GAPDH expression.

### 5.18 Bone Marrow‐derived Macrophage (BMDM) Cultures

Bone marrow cells were flushed and isolated from femurs and tibias of healthy C57BL/6 mice (7‐8 weeks old). Macrophage precursors were cultured and differentiated into macrophages for 7 days in Mouse M‐CSF Recombinant Protein (M‐CSF, 315‐02‐10UG, PeproTech, USA)‐conditioned Dulbecco's Modified Eagle Medium (DMEM; Gibco, Grand Island, NY, USA) containing 10% FBS and 1% penicillin‐streptomycin (15140122, Gibco, USA).

### 5.19 SPP1 and CD44 Blockade Assay

To block SPP1‐CD44 signaling, BMDMs derived from wild‐type (WT) and TREM2^−^/^−^ (KO) mice were co‐cultured with purified CD8^+^ T cells. BMDMs were pretreated with SPP1 neutralizing antibody (2 µg/mL, AF808, R&D Systems, USA) for 1 h, while CD8^+^ T cells were pretreated with CD44 blocking antibody (6 µg/mL, A2174, Selleck, USA) for 3 h. Following medium replacement, the pretreated BMDMs and CD8^+^ T cells were co‐cultured for 48 h. CD8^+^ T cells were then collected for subsequent flow cytometry and molecular analyses.

### 5.20 siRNA Transfection

TREM2‐targeting siRNA and scrambled control siRNA were synthesized by Sangon Biotech (Shanghai, China). The sense strand sequence of si‐TREM2 was 5′‐CUUAUGACGCCUUGAAGCACU‐3′. Differentiated BMDMs were seeded in 6‐well plates at 5 × 10^5^ cells per well. siRNA (30 nM) and Lipofectamine RNAiMAX (13778150, Thermo Fisher Scientific, USA) were each diluted in Opti‐MEM (Thermo Fisher Scientific, USA), combined, and incubated at room temperature for 20 min to allow complex formation. The transfection complexes were then added to cells. After 60 h of transfection, the medium was replaced with complete DMEM for subsequent experiments. Knockdown efficiency was confirmed by qPCR and western blot.

### 5.21 Autophagic Flux Assay

To assess autophagic flux, BMDMs transfected with siNC or siTREM2 were co‐cultured with CD8^+^ T cells for 48 h. Bafilomycin A1 (BafA1, 100 nM, HY‐100558, MCE, USA) was added during the final 4 h of co‐culture. CD8^+^ T cells were subsequently collected for western blot, flow cytometry, and ATP measurement.

### 5.22 Mouse CD8^+^ T Cells Bag3 Knockdown Assay

Following 60 h activation with anti‐CD3/CD28, primary mouse CD8^+^ T cells were subjected to BAG3 knockdown using the ProteanFect Max Primary T Cell Transfection Kit (PT03, Nanoportal Biotech, UK), according to the manufacturer's protocol. Bag3‐targeting siRNA and scrambled control siRNA were synthesized by Sangon Biotech (Shanghai, China). After 48 h of transfection, the medium was replaced with complete RPMI 1640, and CD8^+^ T cells were co‐cultured with WT or TREM2^−^/^−^ BMDMs for 48 h. Knockdown efficiency was confirmed by qPCR and western blot.

### 5.23 Assessment of Mitochondrial Membrane Potential

Mitochondrial membrane potential was measured using the JC‐1 Mitochondrial Membrane Potential Assay Kit (C2006, Beyotime, China) according to the manufacturer's instructions. Briefly, CD8^+^ T cells were collected after the indicated treatments and incubated with the JC‐1 staining working solution at 37°C for 20 min. Cells were then washed with JC‐1 staining buffer and analyzed by flow cytometry. The mitochondrial membrane potential was evaluated based on the ratio of JC‐1 aggregate fluorescence to monomer fluorescence.

### 5.24 Mitochondrial Superoxide Detection

Mitochondrial superoxide levels were measured using the Mitochondrial Superoxide Indicator Assay Kit (S0061S, Beyotime, China) according to the manufacturer's instructions. Briefly, CD8^+^ T cells were collected after the indicated treatments and incubated with the mitochondrial superoxide detection probe at 37°C for 20 min in the dark. Cells were then washed and analyzed by flow cytometry. Mitochondrial superoxide levels were quantified based on the fluorescence intensity of the probe.

### 5.25 ATP Measurement

Cellular ATP levels were measured using the ATP Assay Kit (S0027, Beyotime, China) according to the manufacturer's instructions. Briefly, CD8^+^ T cells were collected after the indicated treatments and lysed for ATP extraction. ATP levels were determined by measuring luminescence using a microplate reader. Protein concentrations of corresponding samples were determined using the Bradford Protein Assay Kit (Detergent Compatible) (P0006C, Beyotime, China), and ATP levels were normalized to total protein content.

### 5.26 Apoptosis Assay

CD8^+^ T cells were co‐cultured with BMDMs transfected with scrambled control siRNA (siNC) or TREM2‐targeting siRNA (siTREM2) at a ratio of 8:1 (T cells to macrophages) at 37°C for 48 h. In the YM‐1 treatment group, 5 µm YM‐1 was added to the co‐culture system of siTREM2‐transfected BMDMs and CD8^+^ T cells. Apoptosis of CD8^+^ T cells was assessed using the BD Pharmingen FITC Annexin V Apoptosis Detection Kit I (556547, BD Biosciences, USA) according to the manufacturer's instructions. Cells were washed twice with PBS and resuspended in 1× binding buffer. Cells were stained with 5 µL FITC Annexin V and 5 µL 7‐AAD for 15 min at room temperature in the dark. Staining was terminated by adding 400 µL 1× binding buffer, and samples were analyzed by flow cytometry within 1 h.

### 5.27 Statistics and Reproducibility

Data are presented as mean ± SEM. For comparisons between two groups, an unpaired two‐tailed Student's *t*‐test or Mann–Whitney U test was used depending on data normality assessed by the Shapiro–Wilk test. For comparisons among three or more groups, one‐way ANOVA followed by Tukey's multiple comparisons post‐hoc test was applied for normally distributed data, and the Kruskal–Wallis test followed by Dunn's multiple comparisons post‐hoc test was used for non‐normally distributed data. Analysis was performed using GraphPad Prism software, version 10. Statistical significance was defined as ^*^ *p* < 0.05, ^**^ *p* < 0.01, ^***^ *p* < 0.001, ^****^ *p* < 0.0001. Each figure legend denotes the individual *p*‐values for the groups being compared.

## Author Contributions

M.Y. and X.J. conducted all the experiments and arranged the figures and the manuscript. L.X., S.W., and Z.Z. assisted with the animal experiments. Q.R. and N.L. provided technological support for biochemical experiments. X.M. assisted with sample collection. Q. L. assisted with the in vivo neutralizing antibody experiments. G.W. initiated and supervised the project and edited the manuscript. All authors approved the final manuscript.

## Funding

This research was supported by the National Key Research and Development Program of China (2023YFC2705405), Shandong Provincial Natural Science Foundation (ZR2023MH177), the Special Foundation for Taishan Scholars (No. tstp20230657), and the National Natural Science Foundation of China (grant numbers 82371653 and 82301855).

## Conflicts of Interest

The authors declare no conflicts of interest.

## Supporting information




**Supporting File**: advs78385‐sup‐0001‐SuppMat.docx.

## Data Availability

The data that support the findings of this study are available on request from the corresponding author. The data are not publicly available due to privacy or ethical restrictions.
