# Supplementary material for: TREM2+ Macrophages Promote Immune Evasion in Endometriosis Through BAG3‐Associated Regulation of CD8+ T Cell Autophagy
Source: Adv Sci (Weinh). 2026 Nov 12:e78385. Online ahead of print. doi: 10.1002/advs.78385 (PMC13755765; doi:10.1002/advs.78385)
Supplement: Supplementary file 1 [file ADVS-9999-e78385-s001.docx]

# Supplemental Material

**Supplemental Table 1 Characteristics of patients recruited for flow cytometry analysis.**

| **Item** | **Endometriosis(n=52)** | **Controls(n=29)** | **Statistical significance** |
| --- | --- | --- | --- |
| Age(year) | Mean=35.7 | Mean=34.8 |  |
|  | Median=35 | Median=36 | NS |
|  | Range=24-47 | Range=17-54 |  |
| BMI(kg/m^2^) | Mean=23.43 | Mean=24.32 | NS |
|  | Median=21.63 | Median=24.22 |  |
|  | Range=16.41-30.82 | Range=19.72-31.65 |  |
| Menstrual phase |  |  | NS |
| Proliferative | 9 (33.3%) | 6 (40%) |  |
| Secretory | 18(66.7%) | 9(60%) |  |
| Gravidity |  |  | NS |
| 0 | 6(22.2%) | 6(40.0%) |  |
| 1 | 7(25.9%) | 1(6.7%) |  |
| ≥2 | 14(51.9%) | 8(53.3%) |  |
| Miscarriages |  |  | NS |
| 0 | 15(55.6%) | 8(53.3%) |  |
| 1 | 5(18.5%) | 3(20.0%) |  |
| ≥2 | 7(25.9%) | 4(26.7%) |  |

BMI, body mass index; NS, no significance.

**Supplemental Table 2 Flow cytometry fluorochromes used for human.**

| **Antibody** | **Fluorochrome** | **Clone** | **Catalogue no** |
| --- | --- | --- | --- |
| CD14 | BUV563 | M5E2 | BD 741360 |
| CD16 | APC-Cy7 | 3G8 | BD 557788 |
| CD11b | BUV737 | M1/70 | BD 612800 |
| CD1c | BUV615 | F10/21A3 | BD 751476 |
| CD45 | BUV805 | HI30 | Invitrogen 368-0459-42 |
| CD163 | BV421 | GHI/61 | 562643 |
| TREM2 | PE | 237920 | R&D Systems FAB17291P |
| CD206 | BV480 | 19.2 | BD 746279 |
| CD86 | BB700 | 2331 | BD 566473 |
| CD68 | PE-Cy7 | Y1/82A | BD 565595 |
| GPNMB | AF594 | 303822 | R&D Systems FAB25501T |
| L/D | FVS440 | N/A | BD 566332 |
| CD3E | BUV737 | UCHT1 | BD 612750 |
| CD20 | BV480 | L27 | BD 566181 |
| CD4 | BV786 | SK3 | BD 563877 |
| CD8 | PE-Cy7 | HIT8a | BD 300914 |
| CD45 | BUV805 | HI30 | BD 612891 |
| CD56 | APC-R700 | NCAM16.2 | BD 565139 |
| NKP80 | PE | 5D12 | BD 566329 |
| NKG2A | BV711 | 131411 | BD 747919 |
| NKG2C | BB700 | 134591 | BD 748162 |
| Perforin | BV421 | δG9 | Biolegend 308122 |
| GZMB | PE-CF594 | GB11 | BD 562462 |
| Tigit | BUV615 | 741182 | BD 752314 |
| CD25 | BUV661 | M-A251 | BD 741608 |
| CD127 | BB515 | HIL-7R-M21 | BD 564423 |
| CD14 | BUV496 | M5E2 | BD 750381 |
| CD16 | BUV563 | 3G8 | BD 748851 |

**Supplemental Table 3 Flow cytometry fluorochromes used for mouse.**

| **Antibody** | **Fluorochrome** | **Clone** | **Catalogue no** |
| --- | --- | --- | --- |
| CD19b | FITC | ID3 | BD 557398 |
| NK1.1 | BV786 | PK136 | Invitrogen 417-5941-82 |
| CD45 | Percp/Cyanine5.5 | 30-F11 | Biolegend 103131 |
| CD3 | APC-Cy7 | 17A2 | BD 560590 |
| NKG2C | Alexa Fluor 405 | 2098A | R&D Systems FAB1382V |
| NKG2A | BV711 | 16a11 | BD 755253 |
| Tigit | BV650 | 1G9 | BD 744213 |
| CD8 | BV605 | 53-6.7 | Biolegend 100743 |
| Perforin | PE | EBioOMAK-D | Invitrogen 12-9392-82 |
| GZMB | APC | NGZB | Invitrogen 17-8898-82 |
| CD11b | Percp-Cyanine5.5 | M1/70 | Invitrogen 45-0112-82 |
| F4/80 | APC | BM8 | Invitrogen 17-4801-82 |
| TREM | APC | 237920 | R&D Systems FAB17291A |
| L/D | BV421 | N/A | Invitrogen 65-0863-18 |

**Supplementary Table 4** Primer sequences used in qRT-PCR analysis.

| Gene | Primer Sequence (5’–3') | |
| --- | --- | --- |
| GAPDH | Forward | AGGTCGGTGTGAACGGATTTG |
|  | Reverse | TGTAGACCATGTAGTTGAGGTCA |
| Pdcd1 | Forward | ACCCTGGTCATTCACTTGGG |
|  | Reverse | CATTTGCTCCCCTCTGCCTG |
| Bag3 | Forward | TGCCAGTGTCCCTACATCCT |
|  | Reverse | AGGTGGGTCTGCTCCTCAAT |
| Becn1 | Forward | ATGGAGGGGTCTAAGGCGTC |
|  | Reverse | TGGGCTGTGGTAAGTAATGGA |
| Map1lc3b | Forward | CGATACAAGGGGGAGAAGACA |
|  | Reverse | ACTTCGGAGATGGGAGTGGA |
| Trem2 | Forward | CTGGAACCGTCACCATCACTC |
|  | Reverse | CGAAACTCGATGACTCCTCGG |

**Figure S1. Single-cell transcriptomic profiling of the cellular landscape in endometriosis**

**A.** UMAP visualization of single-cell transcriptomic data identifying 16 distinct cell clusters in endometriosis and control tissue samples.

**B.** UMAP feature plots displaying expression of canonical marker genes used for cell-type annotation: CD79A (B cells), CD1C (DC-CD1c), VWF (endothelial cells), DCN (fibroblasts), CD14 (macrophages), TPSAB1 (mast cells), FCGR3B (neutrophils), NKG7 (NK cells), and MYH11 (SMC/pericytes). Color intensity reflects expression level.

**C.** Stacked bar chart showing the relative proportions of each cell type across individual control and endometriosis tissue samples.

**D.** Quantification of immune cell proportions comparing endometriosis and control groups.

**E.** Heatmap of KEGG pathway enrichment analysis across different immune cell clusters, revealing distinct functional pathway signatures for each cell type. Color scale represents enrichment score.

**Figure S2. TREM2^+^ macrophages are markedly enriched in endometriosis.**

**A**. UMAP visualization of macrophages across 11 samples (peritoneal fluid and tissue from endometriosis and control subjects).

**B**. Violin plots displaying expression levels of representative genes across macrophage subsets.

**C**. Heatmap of differentially expressed genes across macrophage subpopulations, highlighting transcriptional distinctions among subsets. Color scale represents relative expression levels.

**D**. Flow cytometric quantification of CD163 and CD206 MFI in macrophages from peritoneal fluid and ectopic tissue of endometriosis and control groups.

**E**. Representative flow cytometry gating strategy for identifying macrophage populations in human peritoneal fluid and tissue.

**F**. UMAP visualization of macrophage subclusters colored by TREM2 expression level (Trem2_mac score), demonstrating that TREM2 expression is concentrated within a distinct macrophage subpopulation.

**Figure S3. CD8⁺ T and NK cells in endometriosis exhibit a functionally exhausted phenotype**

**A.** UMAP visualization of tissue-associated CD8⁺ T cell subpopulations in endometriosis

**B.** Box plots comparing progenitor exhaustion scores of tissue-associated NK cells between endometriosis and control samples.

**C.** Representative flow cytometry gating strategy for identification of CD8⁺ T cells and NK cells in human peritoneal fluid and tissue.

**Figure S4. TREM2⁺ macrophages regulate CD8⁺ T cell function through SPP1-CD44 mediated communication**

A. Representative image and quantification of NKG2A expression on NK cells co-cultured with macrophages from WT or TREM2-knockout (KO) mice (n = 11 per group).
B. Relative mRNA expression of Bag3, Becn1, and Map1lc3b in CD8⁺ T cells co-cultured with WT or KO macrophages (n = 3 per group).

**Figure S5. BAG3 associated autophagy regulation contributes to TREM2⁺ macrophage induced CD8⁺ T cell dysfunction**

A. Representative western blot and quantification of TREM2 in siNC and siTREM2 macrophages (n = 3 per group).
B. B. Relative Trem2 mRNA expression in siNC and siTREM2 macrophages (n = 3 per group).
C. Relative mRNA expression of autophagy-related genes in CD8⁺ T cells co-cultured with siNC, siTREM2, or siTREM2 + YM-1-treated BMDMs (n = 3 per group). Expression normalized to GAPDH.
D. Representative western blots and quantification of BAG3, Beclin-1, and LC3B in CD8⁺ T cells co-cultured with siNC, siTREM2, or siTREM2 + YM-1 macrophages (n = 3 per group).

**E and F**. Representative flow cytometry images and quantification of apoptosis in CD8⁺ T cells co-cultured with siNC, siTREM2, or siTREM2 + YM-1 macrophages (n = 3 per group).

**G**. Representative CFSE image and MFI quantification of CD8⁺ T cell proliferation co-cultured with siNC, siTREM2, or siTREM2 + YM-1 macrophages (n = 3 per group).

**H**. Representative endometriotic lesion image and quantification of lesion weight across three groups that mice were treated with isotype control, isotype + YM-1, or anti-CD8 + YM-1 (n = 3 per group).

**I**. Quantification of GZMB⁺ CD8⁺ T cells (%) in peritoneal fluid of isotype versus isotype + YM-1-treated mice (n = 3 per group).
